# Inequity of maternal-child health services in ASEAN member states from 1993 to 2021

**DOI:** 10.1186/s12939-023-01974-8

**Published:** 2023-08-07

**Authors:** Yikai Feng, Mailikezhati Maimaitiming, Junyi Shi, Minmin Wang, Na Li, Yinzi Jin, Zhi-Jie Zheng

**Affiliations:** 1 Department of Global Health, School of Public Health, Peking University, 38 Xue Yuan Road, Haidian District, Beijing, 100191 China; 2 Institute for Global Health and Development, Peking University, Beijing, China

**Keywords:** Health equity, Maternal-Child Health Services, Southeast Asian

## Abstract

**Introduction:**

Inequity in maternal-child health services is a challenge to global health as it hinders the achievement of Sustainable Development Goals (SDGs) and Universal Health Coverage. Though the Association of Southeast Asian Nations (ASEAN) has made remarkable achievements in maternal-child health, there remain gaps in reaching global goals. This study aimed to compare and investigate the inequity in maternal-child health (MCH) services in ASEAN member states to help guide policy decisions to improve equitable health services in the SDG era and beyond.

**Methods:**

Using the WHO Health Inequality Monitor, we identified inequity summary measures for five MCH services in ASEAN member states from 1993 to 2021: antenatal care, births attended by skilled health personnel, diphtheria, tetanus and pertussis (DTP3) immunization, measles immunization, and polio immunization. We divided the analysis dimension of inequity into urban–rural inequity, economic status inequity, and sub-regional inequity. Trends of absolute and relative inequity in every dimension of MCH services in ASEAN member states were examined with the principal component analysis (PCA).

**Results:**

The mean coverages of MCH services are 98.80% (Thailand), 86.72% (Cambodia), 84.54% (Viet Nam), 78.52 (Indonesia), 76.94% (Timor-Leste), 72.40% (Lao PDR), 68.10% (Philippines) and 48.52% (Myanmar) in 2021. Thailand have the lowest MCH services absolute inequity indexes of -1.945, followed by Vietnam (-1.449). Lao PDR and Myanmar have relatively higher MCH services absolute inequity indexes of 0.852 and 0.054 respectively. The service in Cambodia, Indonesia, and the Philippines is pro-specific regions (with subnational region absolute inequity indexes of -0.02, 0.01, and 1.01 respectively). The service in Myanmar is pro-rich (with economic status absolute inequity index of 0.43). The service in Lao PDR and Timor-Leste is pro-urban areas, pro-rich, and pro-specific regions.

**Conclusion:**

The inequity of MCH services in ASEAN persists but is in a declining trend. Thailand and Vietnam have performed well in ensuring MCH services equity, while Laos and Myanmar are still facing serious inequity dilemmas. The progress of MCH service equity in Myanmar, Cambodia, the Philippines, and Indonesia is uneven. It is acceptable to learn from the successful experiences of Thailand and Vietnam to improve the equities in other ASEAN countries. Policies should be developed according to the specific types of MCH inequity in member states to improve equity levels.

**Supplementary Information:**

The online version contains supplementary material available at 10.1186/s12939-023-01974-8.

## Introduction

Maternal-child health (MCH) is at the core of the United Nations Sustainable Development Goals (SDGs) and universal health coverage (UHC) [1]. Each stage of MCH should be a positive experience, ensuring women and their babies reach their full potential for health and well-being. SDGs 3.1 and 3.2 have made clear requirements for MCH, namely, reducing the global maternal mortality rate (MMR) to less than 70 per 100,000 live births and ending preventable deaths of newborns and children under 5 years old (U5MR) [2]. There are disadvantaged groups (such as those living in poverty, impoverished rural areas, or specific subregions) that do not benefit from economic and health development to the same extent, resulting in increasing inequity in health [3–5]. The maternal mortality ratio, the proportion of mothers that do not survive childbirth, in developing regions is still 14 times higher than in the developed regions. Meanwhile, children in the poorest 20% of the populations are still up to three times more likely to die before their fifth birthday than children in the richest quintile over the world [6–8].

The Association of Southeast Asian Nations (ASEAN), which consists of Negara Brunei Darussalam, the Kingdom of Cambodia, the Republic of Indonesia, the Lao People’s Democratic Republic, Malaysia, the Republic of the Union of Myanmar, the Republic of the Philippines, the Republic of Singapore, the Kingdom of Thailand, the Socialist Republic of Vietnam and the Democratic Republic of Timor-Leste, has accomplished several notable achievements in economic and social development [9]. As the fifth largest economy in the world, ASEAN is characterized by its diversity in terms of geography, society, economic development, and health outcomes. The health healthcare systems and infrastructures vary considerably, which largely contributed to the diverse service delivery status [10]. Although there has been substantial progress in improving the survival and life quality of mothers and children in ASEAN regions, the problem of unequal health services coverage slows the overall progress [11, 12]. From the perspective of service coverage and distribution, ASEAN member states are in rather complicated situations [13], which challenges the formulation of health development and cooperation strategies at the ASEAN level.

For the vision of healthy, caring, and sustainability, the 16^th^ ASEAN Senior Officials Meeting for Health Development (SOMHD) endorsed the ASEAN Post-2015 Health Development Agenda (2021–2025) [14], in which MCH was seen as a health priority under *Health Cluster 3: Strengthening Health System and Access to Care*. Seen from the overall development, the equity of maternal-child health services in ASEAN member states is improving year by year. Some ASEAN member states have made remarkable achievements in MCH services in recent years. For instance, MMR in Laos has decreased from 357.0 in 2012 to 62.0 in 2021, and in the Philippines has decreased from 69.9 in 2013 to 56.8 in 2018. U5MR in Cambodia dropped from 45.2 in 2013 to 25.9 in 2020 [15]. Nevertheless, MCH of ASEAN member states still faces several obstacles. Some national indicators are still far behind the requirements of SDGs. For example, MMR in Cambodia has dropped from 170.0 in 2014 to 141.0 in 2019, but it is still far away from the goal of 70. The overall development of MCH is likely to cover up the fact of unbalanced development, and the unequal distribution of basic health services will eventually slow the progress of health outcomes [16]. Better targeting of policies and resources to subgroups of each country with the greatest need could help to narrow equity gaps and help to achieve the next set of goals [17].

When there are marked inequities, countries which are disadvantaged may lack the resources to participate in the social and economic mainstream of global society [18]. Improving the equity of health services in the ASEAN region is not only the responsibility of ASEAN member states, but also an important vision and working direction of the ASEAN Social and Cultural Community (ASCC) [19]. Health services and health coverage policies after the ASEAN integration should be carefully planned so that every nation can benefit [20]. In previous studies, there has been a lack of discussion on types of inequities in maternal-child health services in ASEAN countries. Describing and comparing the inequities faced by ASEAN member states play important roles in formulating detailed health development and cooperation plans. Inequity ranking can provide a basis for policymakers to choose key countries for assistance and the types of inequity are conducive to the implementation of more precise policy measures and can provide a starting point for subsequent research. The purpose of this study is to analyze the trend of maternal and child health service inequity in ASEAN member states in recent years and find out countries with inequity status using disaggregated data from World Health Organization (WHO). Additionally, we aimed to define factors (urban and rural /rich and poor /uneven regional development) constituting the inequity, and give specific suggestions based on the relevant policies in these countries.

## Data source and methods

### Data source

We obtained data from the Thirteenth General Program of Work (GPW 13) indicators [21] in the WHO Health Inequality Monitor. This dataset contains disaggregated data for indicators used within GPW 13 impact measurement. GPW 13 defines WHO’s strategy for the period 2019–2025 and focuses on measurable impacts on people’s health at the country level [22], gathering from a multitude of sources, such as Demographic and Health Surveys (DHS) and Multiple Indicator Cluster Surveys (MICS). DHS is an ongoing collaboration between the United States Agency for International Development and country-specific agencies. It has collected, analyzed, and disseminated accurate and representative data on population, health, HIV, and nutrition through more than 400 surveys in over 90 countries [23]. MICS carried by UNICEF is one of the largest household survey programs focused on children and women which has covered 116 countries [24].

Since the data set does not contain relevant data of Malaysia, Brunei, and Singapore, this study focused on member states including Cambodia, Indonesia, Laos, the Philippines, Thailand, Timor-Leste, Vietnam, and Myanmar. For this study, the inequity in maternal-child health services was measured by five indicators, including antenatal care coverage, births attended by skilled health personnel, diphtheria, tetanus and pertussis (DTP3) immunization coverage, measles immunization coverage, and polio immunization coverage. Years of data collection ranged from 1993 to 2021. Cambodia has five years of data for 2000, 2005, 2010, 2014, and 2021; Indonesia for 1997, 2002, 2007, 2012, and 2017; Laos for 2011 and 2017; Philippines for 1993, 1998, 2003, 2008, 2013, and 2017; Thailand for 2012, 2015, and 2019. Timor-Leste for 2009 and 2016; Vietnam for 1997, 2002, 2010, 2013, and 2021; Myanmar for 2015.

### Data analysis

For analyzing the degree of inequity by different dimensions robustly, we calculated summary measures including *difference*(X) and *ratio*(Y) of service coverage between urban and rural areas, the richest and poorest wealth quintiles, and areas with the highest and lowest service coverage in each country each year. The *Difference* shows the absolute inequality between two subgroups and the *ratio* shows the relative inequality [25]. The relevant items were sorted out and the evaluation system of maternal-child health service equity was designed, as shown in Appendix Table 1. The evaluation system contains 2 primary indicators (overall absolute/relative inequity index), 6 secondary indicators (absolute/relative inequity index of each dimension), and 30 tertiary indicators (absolute/relative difference of each service between each pair of subgroups).

Then we used the principal component analysis (PCA) to review the trends and types of the inequity in MCH services among ASEAN member states. PCA can classify multiple factors into fewer factors, fully reflect the original information, and the transformed factors have no linear correlation [26]. PCA can extract the main features from the original data, reduce the impact of redundant information, and reduce the dimensions of the original dataset, so as to facilitate data and information visualization and processing. It was first used by Pearson (1901), and then developed and matured by Hotelling (1933), Rao (1964), Cooley & Lohnes (1971), Kshirsagar (1972), Morrison (1976), Gnanadeikan (1977), and Maria, Kent & Bibby (1979) [27–30]. We first carried out PCA and calculated the general scores of secondary indicators in different member states. Then, we obtained the principal component scores of primary indicators in different member states through another round of PCA. We compared and analyzed the changes in indicators and judge the type of inequity of each member state. The Kaiser–Meyer–Olkin (KMO) tests and Bartlett tests were carried out to test whether the data involved is suitable for analysis using PCA. Data analysis was performed using Stata 17.0 (Stata Corp LLC, TX, USA).

## Results

**Fig. 1:**
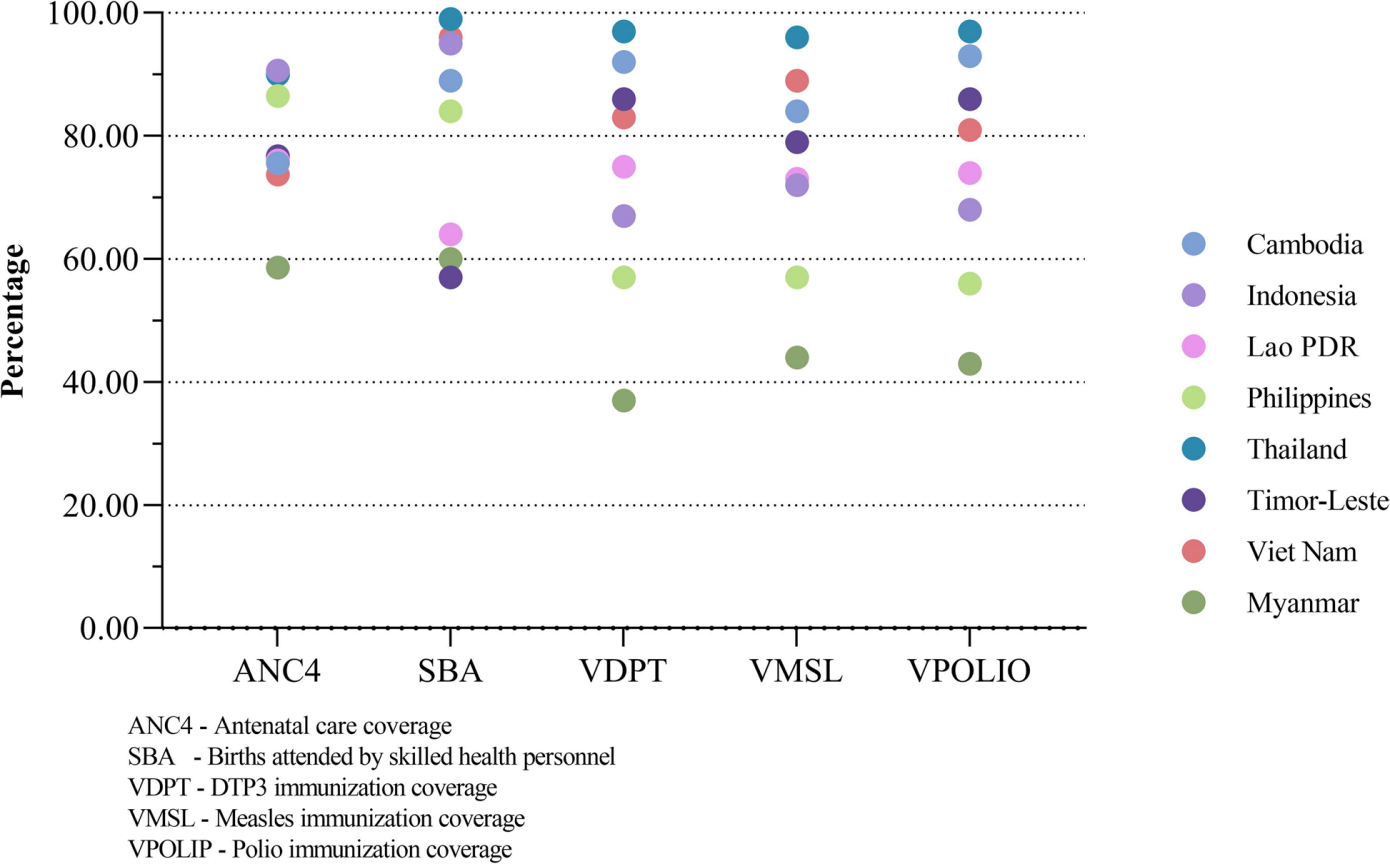
Service coverage of each ASEAN member states

According to the latest data from the WHO Global Health Observatory [31], the coverage of five services among each member state is shown in Fig. 1. In terms of antenatal health care services, Indonesia and Thailand have relatively high coverage of 90.6% and 90.0% respectively, while Myanmar has the lowest coverage of 58.6%. In terms of births attended by skilled health personnel, Thailand has the highest coverage rate of 99%, while Timor-Leste, Myanmar, and Laos have relatively low coverage of 57%, 60%, and 64% respectively. Regarding DTP3 immunization services, Thailand has the highest coverage of 97%, while Myanmar has the lowest coverage of 37%. Regarding measles immunization services, Thailand has the highest coverage of 96%, while Myanmar has the lowest coverage of 44%. Regarding polio immunization services, Thailand has the highest coverage of 97%, while Myanmar has the lowest coverage of 43%. The mean coverages of these services are 98.80% (Thailand), 86.72% (Cambodia), 84.54% (Viet Nam), 78.52 (Indonesia), 76.94% (Timor-Leste), 72.40% (Lao PDR), 68.10% (Philippines) and 48.52% (Myanmar).

**Fig. 2:**
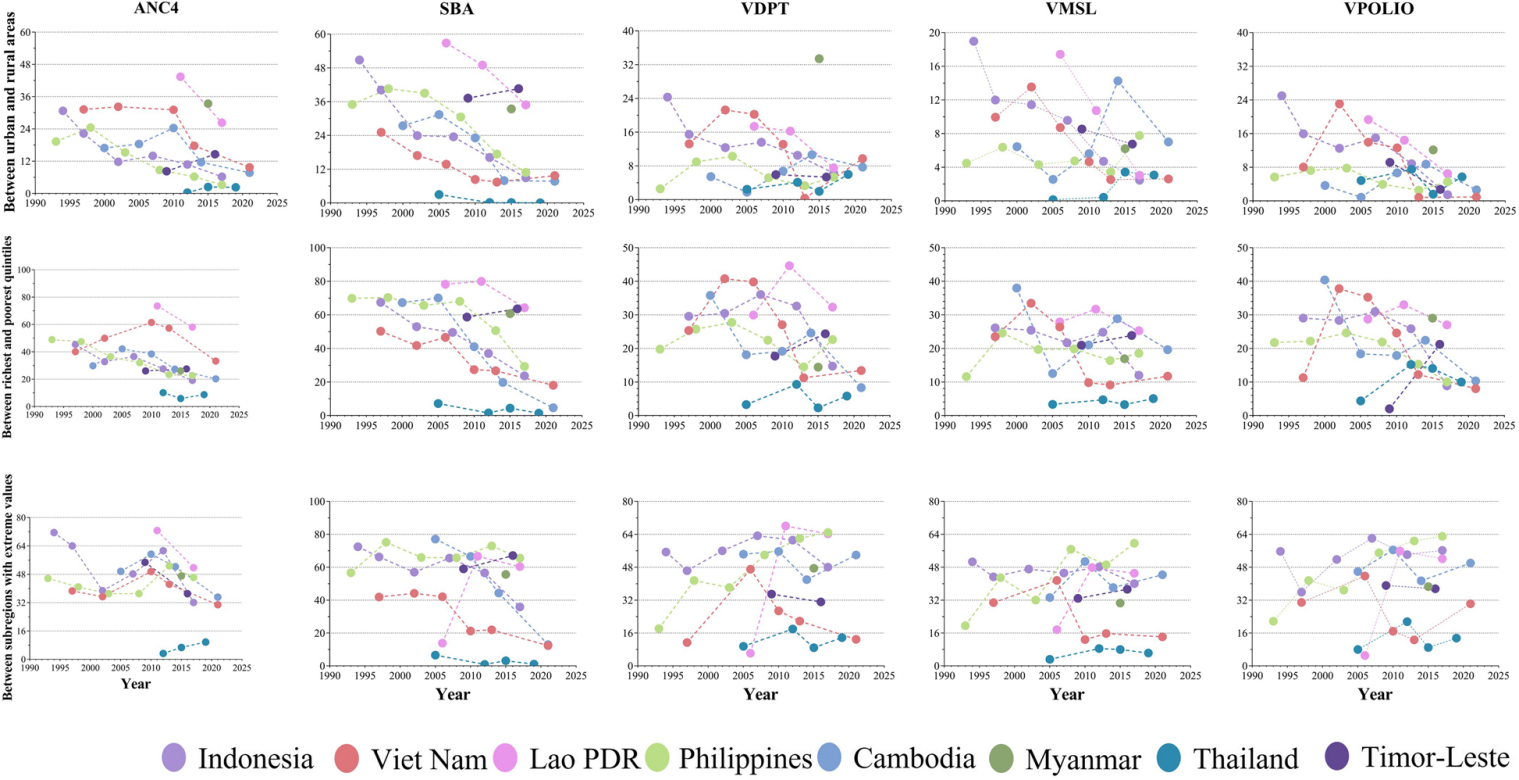
Trend of tertiary indicators (absolute difference) in each country. Note: The vertical column represents the difference in different dimensions of the same service, and the horizontal column represents the difference in different services of the same dimension

Figure 2 shows the trend of the absolute difference of the five services (Appendix Fig. 1 shows the relative difference trend). In terms of urban–rural gap and richest-poorest gap in Cambodia, the indicators generally show a trend of first rising and then declining, while the change in the regional gap is unstable. In terms of Indonesia’s urban–rural gap and richest-poorest gap, the overall indicators show a steady downward trend, while the regional gap has not improved significantly. The indicators of Laos have declined in recent years, but remain at a high level among ASEAN countries. Indicators of Thailand have fluctuated in recent years, but overall they are at a low level. Some indicators of Timor-Leste show a sharp rise. Some indicators in the Philippines have shown a relatively clear upward trend in recent years. After experiencing a significant increase at the end of the last century, various indicators in Vietnam began to decline continuously at the beginning of this century. Since Myanmar only records data for one year, it is not possible to see the trend of change. All indicators of Myanmar are generally at the upper-middle level. Based on the information on the gradual improvement of MCH status in Myanmar provided by the World Bank [32], we speculate that the equity of MCH services in Myanmar is gradually improving.

The KMO values of the absolute difference between urban and rural areas, richest and poorest quintiles, and two subregions with the most extreme values in maternal-child health services are 0.776, 0.733, and 0.816 respectively (0.748, 0.863, and 0.760 respectively for the proportional difference). The significance of the Bartlett spherical test was less than 0.05. The data met the requirements of PCA. The eigenvalues and the proportion of explained variance are obtained after the principal component analysis of the third-level indicators under the second-level indicators. The principal components were selected according to the eigenvalue and proportion to calculate the general scores (Appendix Table 2).

After PCA, the general scores of the secondary indicators of each member state in each year were calculated. The gaps decreased along with the general scores. As shown in Table 1, in general, the scores of secondary indicators of each country show a downward trend over time. The indicators of Cambodia and Indonesia have gradually improved in recent years, but their subnational region inequality indexes are relatively high, which represents that there is still significant inequality between regions. From 2011 to 2017, all indicators in Laos have improved, but its economic status inequality index and subnational region absolute inequality index are still high. According to the information from Myanmar in 2015, the urban–rural absolute inequality index is relatively high among ASEAN member states. The urban–rural inequality index and economic status absolute inequality index in the Philippines has declined, but its subnational region inequality index have become increasingly high, which represents a deterioration in the equality situation between regions. In recent years, three indicators in Thailand have remained at a low level comparing with others, which means it performs well in maintaining service equality across all dimensions. The three indicators of Timor-Leste are all among the relatively high rank. After more than 20 years of development in Vietnam, all indicators have reached a relatively low level.

**Table 1:**
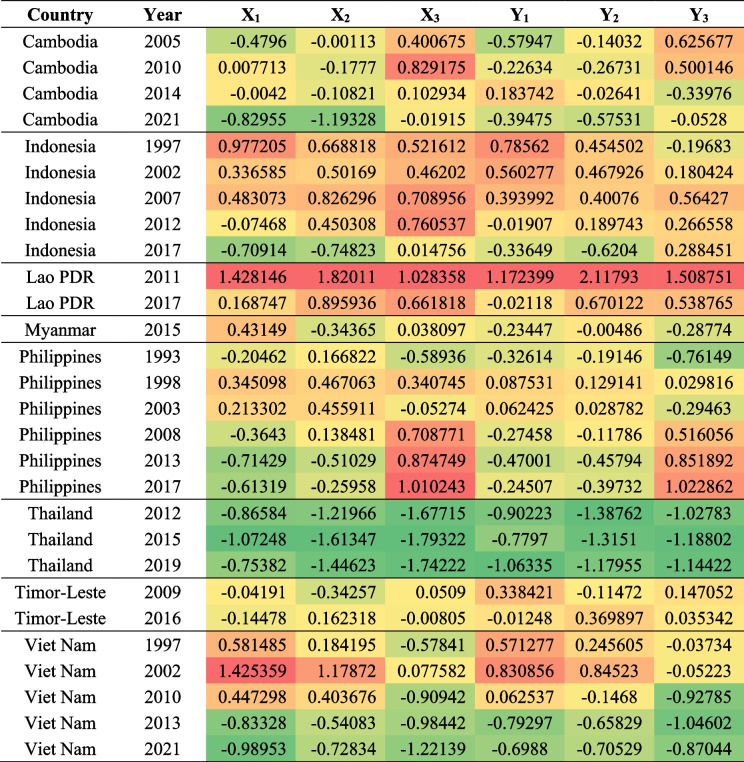
General score of secondary indicators

**Fig. 3:**
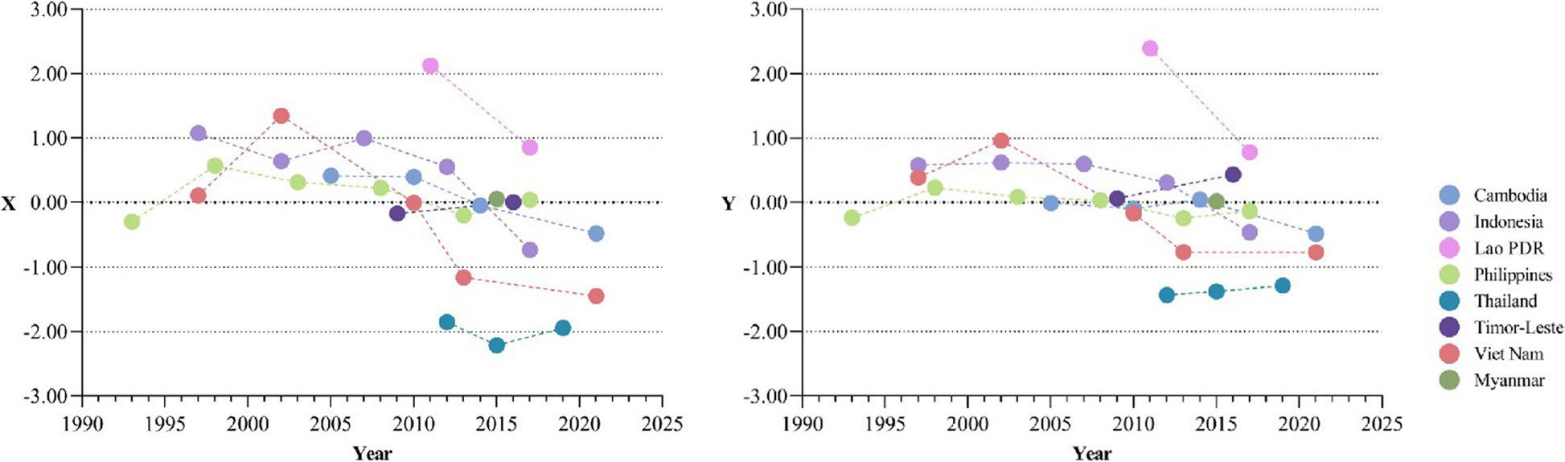
Trend of Maternal-child health services inequity index

Based on the secondary indicators, we conducted PCA again and used the obtained component coefficient to calculate the general score of the primary indicator of maternal and child health services equality to discover the overall situation of inequality in maternal-child health services (Fig. 3) (KMO value was 0.694 and 0.559 respectively for X and Y, Bartlett's significance was all less than 0.05). The lower the score is, the more equal MCH services are. From the perspective of development trends of the MCH services absolute inequality index, there is a reduction in the general score of maternal-child health service absolute/relative inequity in most countries. The general score of Timor-Leste has increased from -0.175 to 0.003, and Indonesia, Viet Nam, and Lao PDR have the most obvious decline trend in recent years. From the absolute number of countries in recent years, a greater equality in MCH services is seen in Thailand with the general score of -1.945, followed by Vietnam (-1.449), Indonesia (-0.732), Cambodia (-0.481), Timor-Leste (0.003), the Philippines (0.038), and Myanmar (0.054). Laos PDR has the highest general score of 0.852. The Maternal-child health services relative inequality index shows similar trends.

Through two rounds of PCA, we obtained the primary indicators and secondary indicators for each country each year with data records. Based on the calculation results of the latest year with data records and the overall coverage of maternal and child health services in various countries, the types of inequities in maternal-child health services in various countries are summarized in Table 2.

**Table 2:** Summary of maternal-child health service coverage information in ASEAN member states

Country	Overall service coverage rank	Service equity rank	Types of inequity in health services	Specific performance
Cambodia	Higher-middle	Higher-middle	High overall-coverage structural inequity	Pro-specific regions
Indonesia	Higher-middle	Higher-middle	High overall-coverage structural inequity	Pro-specific regions
Lao PDR	Lower-middle	Low	Low overall-coverage high inequity	Pro-urban areas/Pro-rich/Pro-specific regions
Philippines	Lower-middle	Lower-middle	Low overall-coverage structural inequity	Pro-specific regions
Thailand	High	High	High overall-coverage high equity	-
Timor-Leste	Lower-middle	Lower-middle	Low overall-coverage high inequality	Pro-urban areas/Pro-rich/Pro-specific regions
Viet Nam	Higher-middle	Higher-middle	High overall-coverage high equity	-
Myanmar	Low	Lower-middle	Low overall-coverage structural inequity	Pro-urban areas

## Discussion

Our results show that there are four types of MCH inequity among ASEAN: (1) high overall-coverage high equity, (2) high overall-coverage structural inequity, (3) low overall-coverage structural inequity, and (4) low overall-coverage high inequity. Thailand and Viet Nam are characterized by type one. Cambodia and Indonesia are characterized by type two. The Philippines and Myanmar are characterized by type three. Lao PDR and Timor-Leste are characterized by type four.

In terms of both coverage and equity level of MCH services, Thailand is a model among ASEAN member states, which is inseparable from its well-established schemes include extensive geographical coverage of functioning primary health care and rural recruitment, home town placement, and financial and non-financial incentives to improve the availability of health workers in underserved areas [33, 34]. In addition, Vietnam's maternal-child health services coverage is at the forefront of ASEAN member states, and its effort to achieve equity has also made considerable progress in recent years, which can in some extent be attributed to the Vietnamese authorities' increasing attention to UHC. In a series of innovative policies of Vietnam's medical and health system in the 1980s, *helping the poor* is one of the important funding and subsidy projects [35]. On March 1, 2016, the Vietnamese government adopted *the Ministry of Health Plan for People’s Health Protection, Care and Promotion 2016–2020* [36], in which a series of indicators were set for evaluation including the implementation of UHC, the integration of services at all levels with primary health care, the reduction of congenital defects and diseases, the increase of access to high-quality reproductive health services, the balance of health human resources at all levels, and the provision of adequate vaccine products at a reasonable price; such content is comprehensive and logical. In recent years, Vietnam has made remarkable achievements in achieving UHC [37]. As one of the low and middle-income countries (LMIC) in ASEAN, its health development-related strategies demand for more in-depth attention and research.

Although the overall service coverage of Cambodia and Indonesia is relatively high, they have inequity in health services in some dimensions. The service coverage in both Cambodia and Indonesia is pro-specific regions. Therefore, while maintaining the overall progress of maternal and child health service coverage, Cambodia and Indonesia should make targeted investments in balancing regional development.

The overall service coverage of the Philippines and Myanmar is relatively low, and they also have inequity in health services in some dimensions. The service coverage in the Philippines is pro-specific regions, and Myanmar is pro-urban areas and pro-specific regions. It is worth noting that the inequity of regional MCH services in the Philippines has shown a worsening trend in recent years. The Philippine government may has recognized this issue [38], and it should make practical efforts (such as providing additional subsidies for health workers in special areas) to balance regional development on the basis of strengthening the comprehensive coverage of MCH services, especially immunization services for children [39]. While the government of Myanmar has endorsed the goal of achieving UHC by 2030 with the aim to improve the health status of the poor and vulnerable, especially women and children, the coverage of maternal-child health services in rural areas and specific regions is relatively unideal. Although the Myanmar authorities have recognized the significance of the availability and distribution of inputs (e.g. human resources, physical infrastructure, supply chain, financial resources) in improving equity and accessibility of health services [40], the political turmoil in Myanmar in recent years has hindered the process of health reform and development to some extent.

The overall level of health service coverage in Lao PDR and Timor-Leste is relatively low, and they have inequity in health services in all dimensions. The current health development plan in Laos was made to solve the cultural, financial, and geographical barriers faced by vulnerable groups in accessing health services, so as to achieve the full coverage of high-quality health services [41]. During its seventh five-year plan, Laos has made considerable achievements in the field of health development. A large number of model health villages have been established, and the health security of pregnant women and children has been improved [42]. Though ranking high among ASEAN member states, the inequity of maternal-child health services in Laos PDR has decreased significantly from 2011 to 2017. As a new member of ASEAN, Timor-Leste has an upward trend in the overall inequity of maternal and child health service coverage, which might be resulted from the increased difference in service coverage between poor and wealthy groups.

It is essential to Enhance the capacity of ASEAN member states, and the focus should be tilted to key countries. In ASEAN Region, five countries – Cambodia, Indonesia, Lao PDR, Myanmar, and the Philippines, remain high in maternal mortality [43]. Timely information disclosure is a powerful tool to support decision-making. A monitoring and evaluation system for MCH equity should be established at the ASEAN level to continuously evaluate the equity of maternal and child intervention measures and their influencing factors [44, 45]. Such a monitoring system requires continuous financial and technical support, so it cannot be separated from the continued political commitment and determination of ASEAN and need close cooperation with all member states.

At the level of ASEAN member states, it is essential to ensure policy decisions do not worsen the status of inequities [46], and to promote operational and resource efficiency to formulate health strategies according to the types of MCH services inequity. The reasons for the inequity in health services between urban and rural areas, the richest and poorest quintiles, and different subregions are different. Additionally, due to the different national conditions and differences in economy, culture, and customs of ASEAN countries, the "universal health coverage" model [47] that emphasizes the provision of unified medical services regardless of the socio-economic status of regions and populations is not necessarily the most effective means to bridge the gap in the coverage rate of maternal and child health interventions in different regions and populations. Targeted research should be carried out based on specific conditions and local context [48] of each country. Benefiting from the fact that after years of development in rural health, the difference in MCH service coverage between urban and rural areas in member states is gradually easing, yet the absolute and relative inequity between urban and rural areas is still a problem worthy of attention for Laos. Thus, the construction of MCH human resources at the rural level in Laos should be further strengthened to improve the health literacy of the rural population. Historically in countries like the Philippines, MCH services utilization has been pro-rich, but pro-poor health policy reforms in the Philippines have expanded health insurance coverage [49] and alleviated the gap between the rich and the poor covered by MCH services.

This study has several limitations. First, WHO Health Inequality Monitor does not contain all ASEAN member states, so comparative analysis can only be conducted among the eight member states with relevant data. Second, to include as many years, countries, and inequity dimensions as possible, we ultimately only selected five MCH indicators for analysis. It is necessary to explore and use more detailed data to conduct more in-depth research on the situation and causes of inequity in maternal and child health services in ASEAN countries in the future, like integrating service quality information based on crude service coverage. Third, the statistical data for some member states have not been updated for several year, meaning that there might be a change in the inequity pattern of maternal-child health services in these countries in recent years. (e.g. The COVID-19 pandemic may have magnified inequities and threatens to exacerbate them) [50].

## Conclusion

Equity analysis is vitally important to identify who gets the worst quality of care, which helps guide policy decisions toward equitable distribution of health resources in the SDG era and beyond [51]. Our findings indicate that inequity in maternal-child health services among ASEAN persists although it is in a declining trend. There are four types of maternal-child health inequity, which means the implementation of targeted measures is needed. To develop best practices and regional approaches to promote health equity in a multi-ethnic, multicultural, and multi-economic region, ASEAN should give full play to its leading role and make a policy commitment to the construction of regional MCH resources and health equity monitoring network, and further explore and promote the relevant experiences of Thailand and Vietnam to enhance the equity of maternal and child health coverage. Policies should be structured and implemented according to different types of maternal and child health inequity. In addition, in order to promote the construction of UHC, research and analysis should also be conducted on inequalities in other areas, including chronic diseases, service capacity and accessibility [52]. This requires richer data support and more diversified analysis methods to assist.

### Supplementary Information

**Additional file 1: Appendix Table 1.** List of maternal and child health service equity indicators. **Appendix Table 2.**  The eigenvalue and contribution proportion of every principal component. **Appendix Figure 1.** Trend of tertiary indicators (relative difference) in each country.

## Data Availability

Data used in this study were downloaded from WHO Health Inequality Monitor (https://www.who.int/data/inequality-monitor/data). This data repository contains datasets of disaggregated data covering diverse topics and dimensions of inequality, from a variety of publicly available data sources.
