# Supplementary material for: Inequity of maternal-child health services in ASEAN member states from 1993 to 2021
Source: Int J Equity Health. 2023 Aug 7;22:149. doi: 10.1186/s12939-023-01974-8 (PMC10408145; doi:10.1186/s12939-023-01974-8)
Supplement: Supplementary file 1 — Additional file 1: Appendix Table 1. List of maternal and child health service equity indicators. Appendix Table 2. The eigenvalue and contribution proportion of every principal component. Appendix Figure 1. Trend of tertiary indicators (relative difference) in each country. [file 12939_2023_1974_MOESM1_ESM.docx]

**Appendix Table. 1 List of maternal and child health service equity indicators**

| **Primary indicator** | **Secondary indicator** | **Tertiary indicator** |
| --- | --- | --- |
| Maternal and child health services absolute inequity index (X） | Urban-rural absolute inequity index in Maternal-child health services（X_1_) | The absolute difference between urban and rural areas in antenatal care coverage（X_11_)^a^ |
|  |  | The absolute difference between urban and rural areas in births attended by skilled health personnel（X_12_)^b^ |
|  |  | The absolute difference between urban and rural areas in DTP3 immunization coverage（X_13_)^c^ |
|  |  | The absolute difference between urban and rural areas in measles immunization coverage（X_14_)^d^ |
|  |  | The absolute difference between urban and rural areas in polio immunization coverage（X_15_)^e^ |
|  | Economic status absolute inequity index in Maternal-child health services（X_2_) | The absolute difference between the richest and poorest quintiles in antenatal care coverage（X_21_) |
|  |  | The absolute difference between the richest and poorest quintiles in births attended by skilled health personnel（X_22_) |
|  |  | The absolute difference between the richest and poorest quintiles in DTP3 immunization coverage（X_23_) |
|  |  | The absolute difference between the richest and poorest quintiles in measles immunization coverage（X_24_) |
|  |  | The absolute difference between the richest and poorest quintiles in polio immunization coverage（X_25_) |
|  | Subnational region absolute inequity index in Maternal-child health services（X_3_) | The absolute difference between the two subregions with the most extreme values in antenatal care coverage（X_31_) |
|  |  | The absolute difference between the two subregions with the most extreme values in births attended by skilled health personnel（X_32_) |
|  |  | The absolute difference between the two subregions with the most extreme values in births attended by skilled health personnel（X_33_) |
|  |  | The absolute difference between the two subregions with the most extreme values in births attended by skilled health personnel（X_34_) |
|  |  | The absolute difference between the two subregions with the most extreme values in births attended by skilled health personnel（X_35_) |
| Maternal and child health services relative inequity index (Y） | Urban-rural relative inequity index in Maternal-child health services（Y_1_) | Proportional difference between urban and rural areas in antenatal care coverage（Y_11_) |
|  |  | Proportional difference between urban and rural areas in births attended by skilled health personnel（Y_12_) |
|  |  | Proportional difference between urban and rural areas in DTP3 immunization coverage（Y_13_) |
|  |  | Proportional difference between urban and rural areas in measles immunization coverage（Y_14_) |
|  |  | Proportional difference between urban and rural areas in polio immunization coverage（Y_15_) |
|  | Economic status relative inequality index in Maternal-child health services（Y_2_) | Proportional difference between richest and poorest quintiles in antenatal care coverage（Y_21_) |
|  |  | Proportional difference between richest and poorest quintiles in births attended by skilled health personnel（Y_22_) |
|  |  | Proportional difference between richest and poorest quintiles in DTP3 immunization coverage（Y_23_) |
|  |  | Proportional difference between richest and poorest quintiles in measles immunization coverage（Y_24_) |
|  |  | Proportional difference between richest and poorest quintiles in polio immunization coverage（Y_25_) |
|  | Subnational region relative inequality index in Maternal-child health services（Y_3_) | Proportional difference between two subregions with the most extreme values in antenatal care coverage（Y_31_) |
|  |  | Proportional difference between the two subregions with the most extreme values in births attended by skilled health personnel（Y_32_) |
|  |  | Proportional difference between the two subregions with the most extreme values in births attended by skilled health personnel（Y_33_) |
|  |  | Proportional difference between the two subregions with the most extreme values in births attended by skilled health personnel（Y_34_) |
|  |  | Proportional difference between the two subregions with the most extreme values in births attended by skilled health personnel（Y_35_) |
| a. Percentage of women aged 15–49 with a live birth in a given time period, attended at least four times during pregnancy by any provider (skilled or unskilled) for reasons related to the pregnancy. | | |
| b. Percentage of live births attended during delivery by skilled health personnel. Skilled health personnel includes doctors, nurses, midwives, and other medically trained personnel as defined according to each country. | | |
| c. The percentage of one-year-olds who have received three doses of the combined diphtheria, tetanus toxoid, and pertussis (DTP3) vaccine in a given year. | | |
| d. The percentage of children aged 12–23 months who have received at least one dose of measles-containing vaccine in a given year. | | |
| e. The percentage of one-year-olds who have received three doses of the polio vaccine in a given year. | | |

**Appendix Table. 2 The eigenvalue and contribution proportion of every principal component**

| **Secondary indicator** | **Factor** | **Eigenvalue** | **Proportion（%）** | **Cumulative（%）** |
| --- | --- | --- | --- | --- |
| Urban-rural absolute inequality index in Maternal and child health services（X_1_) | 1 | 3.43205 | 68.64 | 68.64 |
|  | 2 | 0.87141 | 17.43 | 86.07 |
|  | 3 | 0.42061 | 8.41 | 94.48 |
|  | 4 | 0.20298 | 4.06 | 98.54 |
|  | 5 | 0.07296 | 1.46 | 100.00 |
| Economic status absolute inequality index in Maternal and child health services（X_2_) | 1 | 3.42059 | 68.41 | 68.41 |
|  | 2 | 0.69608 | 13.92 | 82.33 |
|  | 3 | 0.45012 | 9.00 | 91.34 |
|  | 4 | 0.33557 | 6.71 | 98.05 |
|  | 5 | 0.09764 | 1.95 | 100.00 |
| Subnational region absolute inequality index in Maternal and child health services（X_3_) | 1 | 3.98595 | 79.72 | 79.72 |
|  | 2 | 0.58893 | 11.78 | 91.50 |
|  | 3 | 0.26903 | 5.38 | 96.88 |
|  | 4 | 0.10085 | 2.02 | 98.90 |
|  | 5 | 0.05524 | 1.11 | 100.00 |
| Urban-rural relative inequality index in Maternal and child health services（Y_1_) | 1 | 3.19914 | 63.98 | 63.98 |
|  | 2 | 0.82034 | 16.41 | 80.39 |
|  | 3 | 0.700712 | 14.01 | 94.40 |
|  | 4 | 0.208108 | 4.16 | 98.57 |
|  | 5 | 0.0717045 | 1.43 | 100.00 |
| Economic status relative inequality index in Maternal and child health services（Y_2_) | 1 | 3.71154 | 74.23 | 74.23 |
|  | 2 | 0.569734 | 11.39 | 85.63 |
|  | 3 | 0.42986 | 8.60 | 94.22 |
|  | 4 | 0.178119 | 3.56 | 97.79 |
|  | 5 | 0.110745 | 2.21 | 100.00 |
| Subnational region relative inequality index in Maternal and child health services（Y_3_) | 1 | 3.03403 | 60.68 | 60.68 |
|  | 2 | 1.03774 | 20.75 | 81.44 |
|  | 3 | 0.619081 | 12.38 | 93.82 |
|  | 4 | 0.207781 | 4.16 | 97.97 |
|  | 5 | 0.101372 | 2.03 | 100.00 |

Under secondary indicator X_1_, the eigenvalue of the first principal component is 3.43, which can explain the variation of 68.64% of the five tertiary indicators; The eigenvalue of the second principal component is 0.87, which can explain 17.43% of the variation. Select these two principal components to calculate the principal component score. Under secondary indicator X_2_, the eigenvalue of the first principal component is 3.42, which can explain the variation of 68.41% of the five third-level indicators; The eigenvalue of the second principal component is 0.70, which can explain 13.92% of the variation. Select these two principal components to calculate the principal component score. Under secondary indicator X_3_, the eigenvalue of the first principal component is 3.99, which can explain the variation of 79.72% in the five tertiary indicators; The eigenvalue of the second principal component is 0.59, which can explain 11.78% of the variation. Select these two principal components to calculate the principal component score. Under secondary indicator Y_1_, the eigenvalue of the first principal component is 3.20, which can explain the variation of 63.98% in the five tertiary indicators; The eigenvalue of the second principal component is 0.82, which can explain 16.41% of the variation. Select these two principal components to calculate the principal component score. Under secondary indicator Y_2_, the eigenvalue of the first principal component is 3.71, which can explain the variation of 74.23% in the five tertiary indicators; The eigenvalue of the second principal component is 0.57, which can explain 11.39% of the variation. Select these two principal components to calculate the principal component score. Under secondary indicator Y_3_, the eigenvalue of the first principal component is 2.63, which can explain the variation of 52.62% in the five tertiary indicators; The eigenvalue of the second principal component is 1.22, which can explain 24.36% of the variation. Select these two principal components to calculate the principal component score.


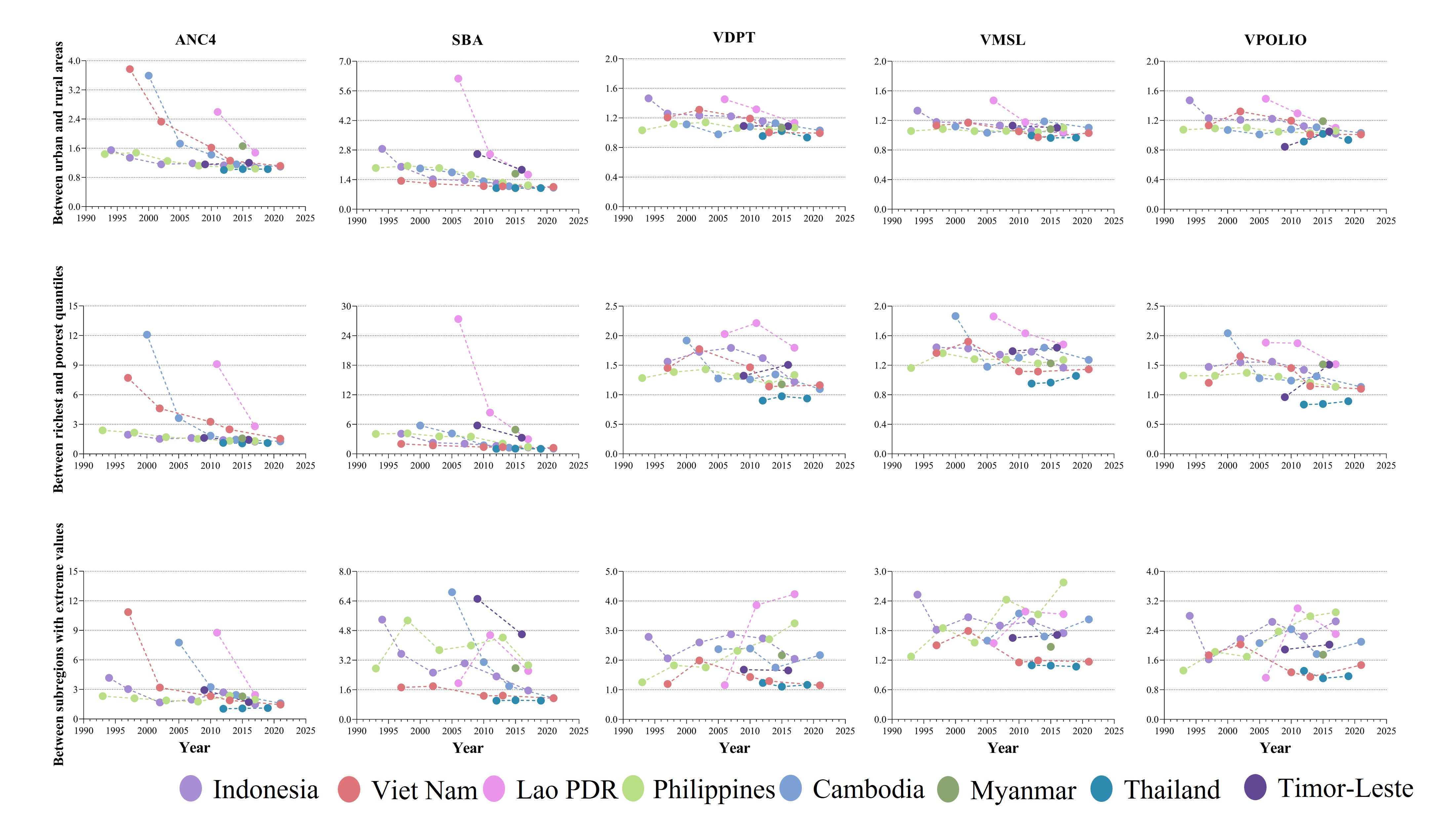


**Appendix Figure 1. Trend of tertiary indicators (relative difference) in each country**
